# Respiratory illness and air pollution from the steel industry: the case of Piquiá de Baixo, Brazil (Preliminary report)

**DOI:** 10.1186/s40248-016-0077-9

**Published:** 2016-11-09

**Authors:** Carla Valenti, Paolo Pozzi, Alessandra Busia, Roberto Mazza, Paolo Bossi, Cinzia De Marco, Ario Alberto Ruprecht, Alessandro Borgini, Roberto Boffi

**Affiliations:** Fondazione IRCCS Istituto Nazionale dei Tumori, Milan, Italy

**Keywords:** Air contaminants, COPD, Fine particle pollution, Lung disease

## Abstract

**Background:**

This report is based on an independent study carried out by medical professionals of the Fondazione IRCCS Istituto Nazionale dei Tumori (National Cancer Institute) in Milan, Italy, and  aimed to assess the incidence of respiratory diseases in a Brazilian community (Piquiá de Baixo, in the city of Açailandia) exposed to extreme air pollution in connection to a local steel manufacturing plant. The study has the objective to contribute to the existing literature on the health risks associated with fine particle pollution (PM2.5) due to steel production with data from Brazil.

**Methods:**

The study is based on a cross-sectional sample of the resident population of Piquiá de Baixo age 16 or over consisting of 220 people. We collected data about the health conditions of participant subjects in two ways: a) medical history questionnaires and b) clinical assessment of respiratory function through spirometry testing. The results were evaluated based on comparative studies.

**Results:**

According to the spirometric tests performed, 28 % of the sample population suffers from respiratory pathologies (for the most part of restrictive rather than obstructive nature). This incidence rate is between six and two times higher than those reported in similar studies carried out in other countries (which range between 4.6 and 14.5 %). In addition, the incidence rate is also significantly high in light of the fact that our sample population did not include the category of subjects most at risk for pulmonary disorders in connection to air pollution caused by the Piquiá steel processing complex: in other words, men and women employed in the steel mills or in connection with their industrial cycle (as many as 434 Piquiá residents age 16 and over were unable to participate to our study due to “work-related reasons”).

**Conclusions:**

In light of the above considerations, we believe that our findings contribute to the existing literature on the correlation between pulmonary disease and air pollution in industrialized areas, while warranting further scientific research on the public health consequences of industrial production in Piquiá de Baixo. In turn, on the ethical plane, we believe that research of this nature strengthens the need to advocate for more severe environmental and health policies aimed at limiting the hazards associated with the steel industry in Piquiá and in similar contexts around the world.

## Background

Thanks to sustained efforts in epidemiological research, we now know that: a) high concentrations of particulate matter (PM) in the air we breathe are the primary factor of health hazards tied to air pollution and b) that the finer the particles are (2.5 micrograms in diameter or smaller), the greater the danger. Most epidemiologists make the case by looking at mortality rates and causes. For example, the American Heart Association has shown time and again that in contexts of environmental pollution due to elevated PM concentrations, people tend to die of respiratory, cardiovascular, and oncological pathologies in greater numbers than normal [1]. Likewise, another important case in the international literature centered on particulate matter with a diameter of 2.5 micrograms (PM 2.5) shows that each concentration increments equal to 10 micrograms per cubic meter (10 μg/m3) corresponds to a 6 % increase in death due to cardio-pulmonary disorders and an 8 % increase in death due to lung cancer [2].

Moreover, research focused specifically on environmental pollution due to industrial production has shown that different industries and manufacturing processes produce particulate matter of different size. When it comes to the steel industry in particular, the literature shows that the processing of iron and other heavy metals, which constitutes the first step of steel manufacturing, produces dust emission with heavy polluting consequences in some area [3–5].

Research focused on industrial pollution from an occupational health standpoint is also of guidance in methodological terms, since it tends to assess risk based on data like respiratory conditions rather than mortality rates. In particular, a number of studies investigating the health hazards tied to occupational dust exposure rely on a combination of medical questionnaires and analysis of lung function through spirometry testing [6, 7].

This approach is especially useful for general population studies based on contexts where death records are either inaccessible or incomplete, for instance due to heavy migration flows. This is precisely the case of our research setting: Piquiá de Baixo, a neighborhood in the city of Açailândia, state of Maranhão, in north-eastern Brazil, where people have shared their vital space with an iron-processing plant tied to the international steel industry since the late 1980s. Faced with resistance from the state and local health board in accessing records and with incomplete information when access was granted, we could not rely on illness and mortality rates to investigate the potential health risks tied to environmental pollution caused by the industrial plant. We thus decided to follow the example of the above-mentioned studies and assess the health conditions of the local population based on medical history questionnaires and the assessment of pulmonary function via spirometry testing, using advanced technology and following the latest international guidelines. The main objective of our investigation is to contribute to the literature on the health risks associated with fine particle pollution (PM 2.5 or smaller) with data from Brazil.

## Methods

### Social and environmental context

The Piquiá de Baixo community, today consisting of about 320 families and 1100 individuals, began taking shape in the 1960s. Close to three decades later, people here suddenly saw a vast area of their neighborhood turn into a major industrial complex tied to the Greater Carajás Program, a government-sponsored project managed by what used to be a national mining corporation, Vale do Rio Doce, today a private multinational company known as Vale S.A. Since its inception in the late 1980s, the project has turned the Carajás region, in the state of Pará, into a massive supply center of iron ore and other minerals destined almost entirely to foreign markets and involving a large area in north-eastern Brazil in terms of transportation and processing, both of which generating significant air pollution. In fact, every day the materials extracted from the Carajás mining complex travel along a 900 km railway, managed by Vale S.A. under a public concession contract, all the way to the São Luís harbor in the neighboring state of Maranhão, where they are then loaded onto cargo ships for export. In addition, about 3 % of the iron ore that travels along the so-called “Carajás corridor” reaches the São Luís harbor in processed form, thanks to exclusive sales agreements between Vale S.A. and companies based in Marabá, Açailândia, and Bacabeira that are specialized in converting raw iron ore into pig iron, the first step of the steel manufacturing cycle. In all three cities, the industrial area generates air pollution both through production and through transportation of material to and from the Carajás railroad.

Açailândia’s industrial area was built right next to the Piquiá de Baixo neighborhood. Today, it consists of five steel plants that include 14 blast furnaces built at the beginning of the Greater Carajás Program in the late 1980s, plus three power plants and one cement plant that were added subsequently. This immense industrial complex sits in close proximity the local public school and private homes: the average distance is 400 m, with the closest homes only 50 m away and sits in close proximity to residential homes.

At the onset of our study, we asked the local and state health board permission to consult community health records, backed by the International Federation of Human Rights (FIDH). However, we found the records to be incomplete and an unreliable source of quantifiable data [8]. There are, however, a number of court-mandated reports and surveys connected with a class action lawsuit that is being carried forth by Piquiá residents against the neighboring industrial complex, which describe the environmental and health hazards associated with the area’s industrial cycle.

In particular, according to an environmental evaluation mandated by the Court of Law of Acailândia in 2007, the “cohabitation” between the industrial complex and the local community is unsustainable for a number of reasons, including the impact of fine particulate matter and gases released by the blast furnaces, which run on a continuous cycle and without the use of chemical filters or gas incinerators [9].

**Fig. 1:**
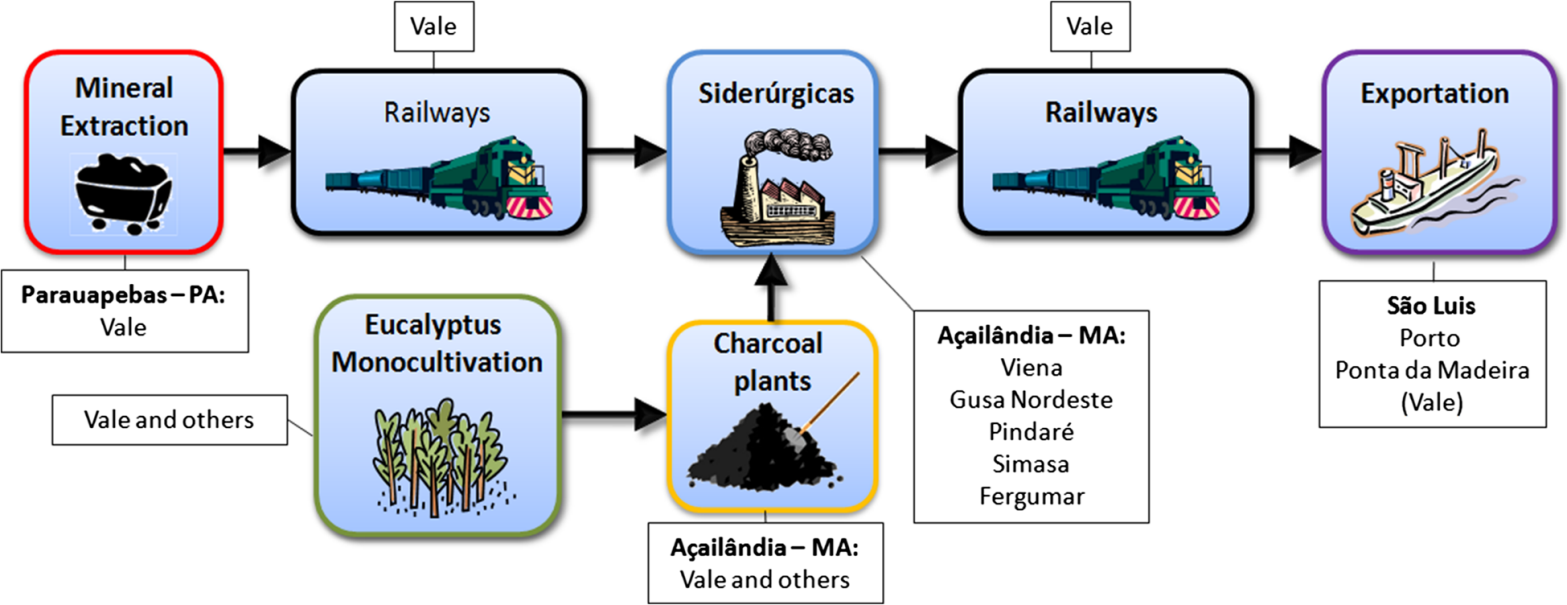
Schematic representation of the cycle of mining, processing and export of iron in Carajás

Besides pollution produced directly by the industrial complex, Piquiá residents incur in further exposure to toxic contaminants in connection to the transportation of raw and processed material along the stretches of the Carajás Railroad and federal highway BR 222 that run alongside their neighborhood. In fact, the railway carries over 300,000 tons of iron ore on a daily basis, while the local road connecting to the highway supports the everyday traffic of dozens of trucks that arrive to the processing plant carrying with charcoal and raw iron ore and leave loaded with either processed iron directed to the railroad or with waste material and dust destined to open air dumps that are scattered throughout the area (Fig. 1).

The health risks associated with air pollution become exponentially dangerous also in light of the socio-economic conditions of the resident population. In fact, over half of the houses in the neighborhood (54 %) are made of wood, which offers limited protection from dust infiltrations and another 12 % are made of mixed materials (wood, mud, and bricks), while brick and stone buildings amount to only 34 % of all homes [10].

Moreover, regardless of the type of construction, all homes are small (60 square meters on average) and have limited ventilation, which causes the dust to be a persistent factor of daily life, outdoor and indoor. Another aggravating factor in terms of respiratory risks is length of exposure to air pollution: 51 % of Piquiá families have been living here for a period ranging from 10 to 40 years and 43 % for a period ranging from 6 to 10 years [9]. Finally, spontaneous relocation is not an option for the majority of residents, considering that 35 % of household heads make less than the Brazilian minimum monthly salary (less than 200 euros) and 38 % makes less than twice this amount [11].

A health assessment report, requested by the Office of Public Defense of the State of Maranhão (Defensoria Pública Estadual del Maranhão) in June of 2011, found 41 % of analyzed subjects to be afflicted by respiratory problems and drew the conclusion that there is a significant correlation between respiratory illness and poor air quality in Piquiá de Baixo [12]. Another survey conducted by the FIDH in 2010 asked residents to fill out self-certified health questionnaires, listing, among other things, their top five health problems. In every case, three out the five problems were of a respiratory nature; specifically, 65 % of participant subjects claimed to suffer from pharyngeal problems, 64 % from cough and mucus secretions, and 53 % from general respiratory issues [13].

### Methodology of the study

The study on which this preliminary report is based aimed to follow up on the above mentioned surveys by assessing the incidence of respiratory pathologies among Piquiá residents in connection to high levels of air pollution generated by the local iron-processing complex.

Data collection consisted in recording the medical history of participant subjects through questionnaires, concentrating on cardiovascular and respiratory pathologies, and assessing respiratory functions through simple spirometry tests, using a MicroLab Carefusion spirometer with Spirometry PC MicroMedical software, according to the latest international guidelines for forced expiratory procedures performed by participant subjects meeting the latest international guidelines for spirometry testing [14].

The study was based on a cross-sectional sample of the Piquiá de Baixo neighborhood population, targeting every household and every person aged 16 or older over a limited period of time (July 15–August 5, 2013).

The study has been approved by the Ethical Committee of the IRCCS Foundation, National Cancer Institute of Milan, Italy. All subjects provided informed consent to the participation; the purpose of the study and the spirometry procedure were explained to all participants with the support of the local community action organization.

The study was divided into two phases. In the first phase, a specialized technician of the IRCCS Foundation/National Cancer Institute of Milan, Italy, met with community organization activists to illustrate the methods of data collection and research objectives. Members of the organization then led the technician from home to home, acting as mediators between her role as investigator and the role of residents as research subjects and assisting in the completion of medical history questionnaires and the performance of spirometry tests.

This first phase accounted for 100 % of questionnaires collected and 70 % of all spirometry tests performed. The remaining 30 % of tests was carried out in the second phase by a local professional nurse. Quality of the data was superimposable between the two assessors.

Values of forced vital capacity (FVC), expiratory volume in the first second (FEV_1_), and Tiffeneau index (FEV_1_/FVC) have been expressed as a percentage of the predicted normal values, taking Brazilian theoretical values as reference [15]. Spirometric data was interpreted with consideration for restrictive abnormalities (simple spirometry tests with FEV_1_/FVC > 0.7 and FVC < 80 %) and obstructive alterations (simple spirometry tests with FEV_1_/FVC < 0.7 and FEV_1_% < 80 %) [16]. The Department of Respiratory Physiopathology of the National Cancer Institute of Milan contributed to data analysis.

## Results

The sample is constituted of 220 subjects out of a target population of 669; exclusion from the study was due to: unavailability for 434 subjects (most of whom were men and mostly due to work reasons), refusal to take the simple spirometry test in the case of 11 subjects, and infective contraindications for four subjects.

The average age was 31 ± 15 years and there was an overall prevalence of women (62 %).

### Spirometry results

Simple spirometry testing of the population sample yielded mean FVC and FEV_1_ values at the limit of the normalcy range (FVC% predicted mean = 90 ± 14 %; FEV_1_% predicted mean = 90 ± 13 %). Results were pathological in the case of 66 out of 220 subjects or 28 % of the sample, subdivided as follows: 65 % of abnormalities were symptomatic of a mild restrictive defect (FVC% predicted mean 72 ± 8 %; FEV_1_% predicted mean 78 ± 10 %), while the other 35 % were compatible with a mild obstructive lung disorder (FVC% predicted mean 87 ± 4 %; FEV_1_% predicted mean = 79 ± 8 %). Expressed as an overall percentage, this means that we found out 19.5 % of restrictive and 10.5 % of obstructive abnormalities, respectively.

### Behavioral risk factors

Cigarette smoke was the first behavioral risk factor we considered when analyzing the results: 20 % of subjects claimed to be an active smoker and 14 % an ex-smoker. Among current smokers, there were slightly more men than women (56 % vs. 44 %) and their mean age was higher than that of the entire population sample (man age: 41 ± 13 years). In terms of frequency, 66 % of smokers in the sample claimed to smoke less than ten cigarettes a day, 27 % between 11 and 20 cigarettes, and 7 % over 40 cigarettes a day.

The second factor we weighed when analyzing spirometry test results was exposure to smoke from charcoal stoves as the source of cooking heat in the neighborhood. We found that in most homes the stove is kept on outdoor porches that are roofed but open on the sides, precisely to ensure ventilation.

The characteristics of exposure to risk factors that could concur in spirometric abnormalities are reported in Table 1.

**Table 1:** Risk factors for the alteration of spirometry indexes

	Years of residence in Piquiá		Exposure to cigarette smoke	Use of charcoal stoves	Respiratory comorbidity
	1–10 years	>10 years			
Male (M)	24 %	76 %	56 %	69 %	74 %
Female (F)	15 %	85 %	44 %	69 %	76 %

## Discussion

The spirometric testing of lung functions carried out on a sample population of 220 Piquia residents - a community in Brazil known to be exposed to high levels of air pollution produced by a local iron processing complex- yielded abnormal results in 28 % of the sample. We consider this to be an extremely significant percentage from a medical standpoint, both in terms of sample demographics and compared to results found in similar studies.

In fact, our sample represented a cohort of young adults (mean age: 31 years old), an age group that generally reports very low rates of spirometric abnormalities, among which the percentage of smokers was comparable to the national average for Brazil [17].

In comparative terms, the European Community Respiratory Health Survey (ECRHS) is a significant reference. The study was carried out in the early 1990s based on general population samples in the 20-44-year-old age group in Europe and several non-European countries. According to the study, the prevalence of diagnosed bronchial asthma ranged from 2 % in Estonia to 11.9 % in Australia [18]. More recent data from the USA revealed a 2.6 % incidence of such as chronic obstructive pulmonary disease (COPD) in the 18-34-year-old age group [19]. If we sum the older ECRHS data with the more recent USA data, we get an age group comparable to that of our study (18 to 44), in which obstructive or mixed alterations of respiratory functions ranges from 4.6 to 14.5 % – well below the 28 % incidence rate we found.

The prevalence of spirometric abnormalities yielded by our study remains significantly high also when compared to USA national data focused on the idiopathic pulmonary fibrosis. In fact, among rare respiratory diseases, idiopathic pulmonary fibrosis is the most common associated with a restrictive spirometric footprint. According to Raghu et al. [20], cases of the pathology diagnosed based on restrictive criteria range between less than 0,005 % and less than 0,028 % (from 14 to 27.9 every 100,000 residents). A limitation of our study lies in not having measured the static lung volumes and there are disputes about the spirometric classification of respiratory alterations to restrictive footprint rather than obstructive [21].

The significance of spirometric abnormalities associated with disorders of a restrictive kind in the cohort of Piquià residents we analyzed (19.5 % of the sample) is especially difficult to explain, since to date there are no published epidemiological studies from Brazil focused on restrictive lung disorders. However, we have grounds to believe that it is strongly correlated with the situation of heavy air pollution caused by the industrial complex. In fact, the presence of fine particles in the air we breathe is associated with a significant drop in respiratory function; and the air quality in Piquiá de Baixo is compromised by the emissions of fourteen blast furnaces that run on continuous cycle without filtering, plus the general polluting effects of the overall production cycle revolving around the local industrial complex.

As regards fine particle pollution a recent study entitled ESCAPE (European Study of Cohorts for Air Pollution Effects) found that every 10 microgram increment per square meter (10 μg/m^3^) in the concentration of PM10 corresponded to a significant drop in the respiratory function of test subjects, quantified as follows: a 44.6 ml decrease in FEV_1_ and a 59.0 ml decrease in FVC, both expressed as absolute value [22]. Recently, another study showed how exposure to ambient air pollution is associated with restrictive ventilatory patterns [23].

As regards spirometric abnormalities associated with obstructive respiratory defects in our population sample, we can advance a more accurate interpretation based on regional and national data for pathologies of this kind. The rate of obstructive abnormalities we found (10.5 %) is close to the one found among Maranhão adolescents, which is 12.7 % [24], and to the national average incidence of asthma in Brazil, which is 8.5 % (albeit with significant differences between the south, 12.6 %, and the north-east, 4.4 %) [25]. In addition, high concentrations of PM10 have been proven to be strongly correlated to increases in hospitalization due to asthma attacks also in Brazil [26].

We acknowledge the limitations of our study, beginning with the limited representativeness of the sample, given its small size. This was mainly due to the impossibility to collect sufficient data from adult male residents due to work-related reasons: as the demographic group with greater employment opportunities, in fact, most men were unavailable for programmed home visits and spirometry test sessions. In light of the fact that employment rates in the community are higher among men than women and that many residents work in the iron processing complex or in connection with it, exposure to polluting substances deriving from iron processing is potentially higher precisely among men; in other words the demographic group for which our study is poor in data. This means that the prevalence of spirometric abnormalities and therefore of respiratory problems in the neighborhood population could be even higher than recorded.

We are also aware that our data does not support direct conclusions on a direct cause-effect relationship between pollution generated from Piquià’s iron-processing plant and the spirometric abnormalities recorded in the local population. In fact, there are various causes that could concur with the pulmonary problems we documented, like the presence of other sources of air pollution, namely fine particles issuing from the trucks that transit on the highway junction road carrying raw and processed material, and the socio-economic context of the local population, as proven by studies that correlate poverty to lower respiratory development [27].

Except for this last potential causal factor, however, we note that both the pollutants produced by blast furnace emissions and those released by the transportation of raw and processed materials to and from the Piquià industrial complex are connected to the presence of steel companies in the area. In a logic of prevention, we thus think it is of fundamental importance to assess, address, and remove all causes of fine particle pollution in Piquia.

Moreover, we would like to point out that our study relies on spirometric abnormalities to evaluate the effects of industrial pollution on pulmonary functions and does not consider the potential correlation between fine particle pollution in the area and higher than normal incidence values of cancer pathologies in general and lung cancer in particular among the local population. Unfortunately, we could not inquire into this cause-effect relationship in the population under analysis, due to lack of specific records in the local health system regarding the incidence of lung cancer. However, given that several studies correlate the annual PM average to the incidence of lung cancer [2, 28], this correlation is likely to exist in Piquià as well.

In closing, the clinical data here reported, the lack of inspections and epidemiological records on the part of the public health system, and the particular environmental characteristics of the Piquia area documented by several court-mandated reports and surveys, lead us to define the Piquià case as emblematic of how the lack of health and environmental policies targeting industrial production results in severe impact on the health conditions of populations that reside in close proximity to processing plants tied to the steel industry.

## Conclusions

This independent study suggests that air quality in Piquiá de Baixo is likely to have a relevant impact on the respiratory health of local residents. Aside from the significant rate of lung function abnormalities we have recorded, research on the health conditions of populations that reside next to industrial steel processing plants alone justifies the need for urgent action to protect Piquiá de Baixo residents from the extremely high levels of pollution they are exposed to. Along with an immediate emergency response to redress the health hazards that threaten men, women, and children in the area (such as collective relocation) we believe that further action should be taken a) to improve the standards of industrial production in the Piquia industrial area, to reduce the effects of fine particle pollution and b) to raise greater awareness in this region of the Maranhão state regarding the connection between air quality and the health conditions of local residents and steel workers, following the example of similar programs implemented elsewhere in Brazil and South America [29].

## Abbreviations

COPD: Chronic obstructive pulmonary disease
ECRHS: European Community Respiratory Health Survey
ESCAPE: European Study of Cohorts for Air Pollution Effects
FEV_1_: Fraction of expiratory volume in the first second
FIDH: Federation of Human Rights
FVC: Forced vital capacity
GOLD: Global initiative for chronic obstructive lung disease
PM: Particulate matter
